# Profiling the MMP family genes as prognostic biomarkers in oral squamous cell carcinoma: from computational discovery to clinical validation in a South Indian cohort

**DOI:** 10.3389/fbinf.2026.1955795

**Published:** 2026-10-16

**Authors:** P. Sakthi Subhashini, T. Bharanidharan, D. Suresh Kumar, S. Asha Devi

**Affiliations:** 1 School of Bio Sciences and Technology, Vellore Institute of Technology, Vellore, Tamil Nadu, India; 2 Department of Surgical Oncology, Center for Oncology, Government Royapettah Hospital, Chennai, Tamil Nadu, India

**Keywords:** differentially expressed genes, ELISA, matrix metalloproteinases, oral squamous cell carcinoma, serum biomarkers

## Abstract

**Background:**

Over the past decade, lifestyle-related habits and environmental exposures have substantially increased the global incidence of Oral squamous cell carcinoma (OSCC). This trend underscores the importance of early screening to improve patient treatment outcomes, as delayed diagnosis severely reduces survival chances. Matrix metalloproteinases (MMPs) are a family of zinc- and calcium-dependent proteolytic enzymes that degrade the components of extracellular matrix, thereby promoting tumor progression, invasion and metastasis. Despite their well-established roles in OSCC, the overall clinical relevance of MMPs remains unclear.

**Methods:**

This study systematically investigates the transcriptional expression landscape of all 23 human MMPs to determine their prognostic relevance in OSCC. Through an integrated analysis of the Gene Expression Omnibus and The Cancer Genome Atlas databases, six overlapping differentially expressed genes of MMP family were identified from the human oral tissue transcriptomic datasets. Prognostic relevance of these MMPs were examined by receiver operating characteristic curves, survival analyses, and predictive nomograms.

**Results:**

Computational analysis revealed that the prediction model achieved optimal accuracy when the six selected MMPs (MMP-1, MMP-10, MMP-3, MMP-12, MMP-13, and MMP-9) were combined with Tumor-Node-Metastasis staging system. Gene Set Enrichment Analysis demonstrated that the MMPs were associated with metabolic reprogramming and proliferation-related signaling pathways, highlighting their contribution towards oral tumor progression. To validate the *in silico* transcriptomic findings, we performed an enzyme-linked immunosorbent assay to quantify the serum levels of MMP-1, MMP-10, and MMP-9 in South Indian OSCC patients. These proteins were significantly elevated in OSCC patients than healthy control samples (*p* < 0.0001, *p* < 0.0001 and *p* = 0.0002) respectively.

**Conclusion:**

This study reveals that the elevated serum levels of MMP-1, MMP- 10 and MMP-9 can serve as minimally invasive prognostic biomarkers for OSCC in the South Indian cohort.

## 1 Introduction

Cancer is a multi-factorial, non-communicable, and heterogenous genetic disease that represents a substantial global health burden (Yang et al., 2019; Jena et al., 2024). According to Global Cancer Observatory reports, 2022, the total incidence of all cancer types in Asia among male and female population is 49.2% (9,826,539) (Ferlay et al., 2024). Amidst a rising global cancer burden, lip and oral cavity malignancies remain disproportionately high in Asia, which accounts for 55.6% (79,979) of global cases and 56.5% (143,759) of related deaths. Cancer of the lip and oral cavity are collectively termed as oral cancer (OC), affecting specific anatomical regions such as tongue, mucosa of the cheek, lips, and oral floor (Miranda-filho and Bray, 2020). Almost 90% of oral tumor originates from the squamous epithelial cells and is typically defined as Oral squamous cell carcinoma (OSCC) (Rahadiani et al., 2023). The worldwide impact of OSCC is increasing due to influence of multiple risk factors including, active tobacco use, alcohol intake, smoking, habitual betel quid chewing, inadequate oral care, malnutrition, human papillomavirus infection, weakened immune system, and excessive sun exposure (Herrero et al., 2003; Ray et al., 2013; Muthu et al., 2018; Kolegova et al., 2025). Among these potential determinants, excessive use of tobacco products, alcohol exposure, and deleterious oral habits contribute to a higher incidence of OSCC among males (46.9%) (Yang et al., 2019) in regions with medium human development index (India: 85.6%, Bangladesh: 8.6%, Myanmar: 1.3%, other countries: 4.5%) (Jena et al., 2024). The primary therapeutic options available to treat OC patients include surgery, chemotherapy, and radiotherapy which are administered based on tumor stage (Thavarool et al., 2019). In spite of standard treatment strategies, major proportion of OSCC cases are diagnosed in advanced stage (metastasis) which contributes to reduced therapeutic response and survival rate (Gangane et al., 2024). Healthcare disparities and delayed diagnosis remains as major contributors to the high mortality and advanced-stage presentation of OSCC in these developing nations (Rutkowska et al., 2020). Therefore, early diagnosis and treatment of OSCC are the two crucial determinants for effective disease management.

Cancer progression is characterized by molecular-level alterations that occur well prior to the emergence of clinical symptoms (Kolegova et al., 2025). Specifically, the extracellular matrix (ECM)-degraders, matrix metalloproteinases (MMPs) are recognized to play potential roles in the early oncogenic alterations such as tumor growth, progression, invasion, migration and metastasis (Anwar and Pratap, 2023). In recent decades, substantial research has targeted the evaluation of oncogenic molecular markers, specifically MMP-2, MMP-3, and MMP-9 that are detectable in bodily-fluids including, plasma, serum, and urine of the patients with cancer (Tadbir et al., 2012; Saini et al., 2025). A total of twenty-three human MMPs (−1, −2, −3, −7, −8, −9, −10, −11, −12, −13, −14, −15, −16, −17, −19, −20, −21, −23B, −24, −25, −26, −27, and −28) have been identified to date and these MMPs are involved in the cleavage of ECM components, such as collagen, fibronectin, elastin, and laminin, thereby weakening the structural integrity of tissues (Visse and Nagase, 2003). The matrix degrading MMPs are synthesised by tumor-derived cells and several components of tumor microenvironment (TME), such as cancer-associated fibroblasts, inflammatory cells, and endothelial cells (Kessenbrock et al., 2010). In specific context of OSCC, these stromal cells secrete cytokines (tumor necrosis factor -α, interleukin family members) and the growth factors (epidermal growth factor, transforming growth factor-β) subsequently upregulate MMP gene expression to accelerate oral tumorigenesis (Li et al., 2024). Increased expression levels of MMP-2 and MMP-9 facilitate degradation of collagen IV that triggers the loss of basement membrane stability, reducing epithelial confinement which creates space for tumor expansion and migration. Such disruption in the structural integrity liberate growth factors that induce tumor progression, angiogenesis, epithelial mesenchymal transition, and oncogenic signaling (Niklander, 2021). Consequently, MMP mediated breakdown facilitates the intravasation of malignant cells into lymphatic vasculature which results in metastatic spread from the primary oral cavity tumor to regional lymph nodes and distant sites. Consistent with these findings, Saini et al. (2025) also proved that the elevated levels of MMP-1, MMP-2, and MMP-9 strongly correlates with metastatic progression in OSCC (Saini et al., 2025). Despite their recognized roles in OSCC, no published literature to date has systematically evaluated the collective influence of all identified human MMPs on cancer progression.

In recent years, computational approaches have been extensively utilised to identify potential therapeutic drug candidates for cancer prevention and management (Lucas et al., 2014; Walsh et al., 2017). Multiple researchers have explored the tissue-level expression patterns and immunohistochemical profiles of MMPs, but few studies have evaluated their corresponding serum levels in Indian OSCC cases (Patel et al., 2007; Benitha et al., 2023; Saini et al., 2024). Concurrently, profiling the circulating levels of multiple MMP family members remains unexplored in the South Indian population. To bridge this gap, the present study employed an integrated bioinformatics approach to systematically identify human MMP family genes and evaluate their prognostic significance. Subsequently, these computational findings were clinically validated using serum samples from South Indian OSCC patients to assess their diagnostic potential. For which, transcriptomic profiles were retrieved from publicly accessible data repositories such as, Gene Expression Omnibus (GEO) and The Cancer Genome Atlas (TCGA) that possess clinical data and the gene expression patterns of OSCC patients. Differentially expressed genes (DEGs) in OSCC tumor tissues were sourced from the two above mentioned databases. Following this, we selected the differentially expressed MMPs to evaluate their suitability as robust predictive and diagnostic biomarkers among OSCC cohort. Taken together, findings from the integrated *in silico* and experimental validation may provide insights into the potential utility of MMPs as serum-based biomarkers for OSCC detection.

## 2 Materials and methods

### 2.1 GEO dataset identification and selection

Gene expression profiles associated with OSCC were retrieved from National Center for Biotechnology Information (NCBI) GEO database (Barrett et al., 2005). From an initial pool of 21 datasets, 12 were selected for analysis based on specific inclusion criteria. The datasets which included at least a minimum of five control samples were considered, additionally the samples marked with clear site of tissue isolation were only taken into study consideration (Table 1). To strengthen our analysis, we also obtained RNA-seq data from OSCC samples within the TCGA-HNSCC cohort (Weinstein et al., 2013). The TCGA-OSCC dataset included 258 tumor samples, eight normal control samples by restricting anatomical site selection to “Base of tongue”, “Floor of mouth”, “Mouth”, “Tongue”, “Lip”, “Gum” and “Hard palate.

**TABLE 1:** Screened dataset from the transcriptomic analysis.

S. No	Datasets	Platform	Number of samples	
			Normal	Tumor
1	GSE10121	DKFZ/Operon human oligo set v4.0	6	35
2	GSE107591	Affymetrix	23	24
3	GSE13601	Affymetrix	26	31
4	GSE138206	Affymetrix	6	6
5	GSE23558	Agilent	5	27
6	GSE25099	Affymetrix	22	57
7	GSE30784	Affymetrix	45	167
8	GSE35261	Operon human 37.6 K V4.0.1 oparray	6	6
9	GSE51010	Affymetrix	8	48
10	GSE9844	Affymetrix	12	26
11	GSE74530	Affymetrix	6	6
12	GSE6791	Affymetrix	14	30
13	TCGA		18	258

### 2.2 Differential gene expression analysis

Expression data from 12 microarray datasets obtained from NCBI’s GEO were processed using limma package in R (v4.2.5) (Ritchie et al., 2015). Within the TCGA cohort, differential expression was assessed via the edgeR package (v4.10) (Robinson et al., 2009). To ensure cross-dataset consistency, multiple microarray platforms or different versions were used (e.g., GSE51010), batch-effect correction was executed using SVA package (v3.61.0) (Leek et al., 2012). The DEGs were considered as significant based on two criteria: an adjusted *p*-value <0.05 and Fold change (FC) > 2. The differentially expressed MMP family members meeting these criteria were selected for further analysis. Expression profiles of MMPs across all 13 datasets (12 GEO + TCGA) were visualized using heatmaps. Further, the shared MMPs across datasets were represented using an UpSet plot.

### 2.3 Survival analysis and prognostic modeling

Clinicopathological metadata associated with the TCGA samples enabled survival modeling using univariate and multivariate Cox regression. These analyses were implemented using survival (v3.8–9) and survminer R packages (v0.5.2). Both clinical and epidemiological parameters considered in this study includes: age (≤60 vs. >60 years), gender (male, female), grade of tumor (G1,G2,G3), Tumor-node-metastasis (TNM) staging (stage I-II vs. stage III-IV), size of tumor (T1-T2 vs. T3-T4), nodal involvement (N0 vs. N1-N2), tumor site (upper: hard palate, upper gum, lip; middle: tongue, cheek mucosa, oral cavity, gum, ventral tongue; lower: base of tongue, floor of mouth, anterior floor, mandible), and lifestyle factors (tobacco and alcohol use). The patient characteristics with *p* value <0.3 in univariate Cox regression model were further selected and subjected to multivariate Cox regression. Pearson correlation analysis was employed to examine the relationship between significantly differentially expressed MMPs. These MMPs were then integrated into a prognostic model, where patient-specific risk score was computed using the equation:

$$\sum_{i=1}^{n}\;{Coef}_{i\;}\times {\mathrm{Exp}}_{i}$$

where *n* represents total number of prognostic genes, the *Coef* denotes regression coefficient, while *Exp* corresponds to gene expression value. Using the median value of risk score as cutoff, OSCC patients were stratified as low- and high-risk groups, whereas Kaplan-Meier (K-M) survival graphs were plotted to compare overall survival (OS) outcomes. Additionally, the TNM staging based survival prediction was also compared using the receiver operating characteristic (ROC) and K-M plots. According to the results of multivariate Cox regression analysis, a nomogram was generated utilizing glm and regplot R packages (v1.1) for the prediction of individualized survival probabilities. Predictive performance was evaluated using concordance index (C-index) and area under the ROC curve (AUC) value. Furthermore, the calibration graphs were plotted to visually assess the performance of prognostic nomogram.

### 2.4 Gene set enrichment analysis (GSEA)

To investigate the biological differences between low- and high-risk OSCC patient groups, GSEA was performed on TCGA tumor samples (Liberzon et al., 2015). For this analysis, the gene sets were obtained from Molecular Signatures Database (MSigDB), particularly using H: hallmark gene sets, C2: Kyoto Encylopedia of Genes and Genomes (KEGG) - derived gene sets, and C6: oncogenic signatures. GSEA was performed using the clusterProfiler (v4.20.0), msigdbr (v26.1.1), enrichplot (v1.30.5), org.Hs.eg.db (v3.23.1), AnnotationDbi (v1.74.0), and dplyr packages (v1.2.1). Pathways characterized by a normalized enrichment score (NES) > 0 were classified as upregulated in high-risk group, whereas those with an NES <0 were considered downregulated.

### 2.5 Sample collection and quantification of MMP-1, MMP-10, and MMP-9 proteins

Computational analyses revealed that six top-most MMPs (MMP-1, MMP -10, MMP-3, MMP-12, MMP-13, and MMP-9) were significantly upregulated in OSCC tissues across the analyzed datasets. From this group, MMP-1, MMP-10, and MMP-9 were selected for downstream evaluation in serum of OSCC patients. This prioritization was based on their consistent manifestation across multiple datasets; specifically, MMP-1 and MMP-10 exhibited the highest cross-dataset overlap appearing in 13 and 12 datasets, respectively, while MMP-9 demonstrated a moderate yet stable presence across 7 datasets, highlighting their biological relevance and justifying their inclusion for experimental validation. Despite the consistent upregulation of MMP-3, MMP-12, and MMP-13, we prioritized MMP-1, MMP-10, and MMP-9 for downstream serum validation due to their documented roles in OSCC progression and high potential as circulating biomarkers. To evaluate the serum protein concentrations of three target MMPs (MMP-1, MMP-10, and MMP-9), we recruited 170 participants from the Department of Surgical Oncology at Government Royapettah Hospital in Chennai, Tamil Nadu, India. This cohort included 79 OSCC patients and 91 disease-free individuals serving as controls. The study obtained ethical approval from the Institutional Ethics Committee of Government Kilpauk Medical College, Chennai, Tamil Nadu, India (ECR/1385/Inst/TN/2020/1341/2024). For the OSCC cohort, inclusion criteria were restricted to patients with histologically confirmed, newly diagnosed, and untreated disease presenting with comprehensive clinicopathological records. Exclusion criteria included previous cancer treatments, viral infections (including HPV), secondary malignancies, or hemolyzed serum samples. The control group consisted of age- and ethnicity-matched healthy individuals free from malignancies or conditions capable of altering serum biomarker profiles. From all these study participants, 3 mL of fresh blood samples were collected in clot-activator tubes. All participants provided written informed consent to participate in this study. The serum fraction was isolated from each of the clotted blood samples (non-haemolysed) upon cold centrifugation conditions (4 °C) at 4,500 rpm for 10 min. The isolated serum samples were carefully transferred into 1.5 mL centrifuge tubes and preserved at −80 °C to prevent degradation until analysis. The protein concentration of three target MMPs (MMP-1, MMP-10, and MMP-9) in serum samples of OSCC cases and disease-free controls were measured using sandwich enzyme-linked immunosorbent assay (ELISA), as per the instructions provided by CUSABIO ELISA kits (MMP-1: CSB-E04672 h, MMP-10: CSB-E04673 h, and MMP-9: CSB-E08006 h). The optical density measurements were obtained at a wavelength of 450 nm with an ELISA plate reader (Bio-Rad xMark™ Microplate Absorbance Spectrophotometer). All the serum samples were analyzed in triplicates, and the unknown protein levels of serum MMP-1, MMP-10, and MMP-9 were calculated from ELISA standard curves.

### 2.6 Statistical analysis

Statistical evaluation of the serum concentration for the three target MMP proteins (MMP-1, MMP-10, and MMP-9) were performed using a two-tailed Mann-Whitney U test, with *p* < 0.01 considered statistically significant. Additionally, the ROC curve analysis was also performed to assess the diagnostic performance of target MMPs, distinguishing OSCC patients from healthy controls. All the statistical analyses were carried out using the GraphPad Prism software (v11.0).

## 3 Results

### 3.1 Identification of differentially expressed MMPs in OSCC

DEGs were screened by comparing OSCC tumor tissues with normal control tissues across the 13 independent datasets (12 GEO + TCGA). From the identified DEGs, MMP family genes were screened, based on threshold of FC > 2 and with a corrected *p*-value <0.05. The gene expression patterns of the MMP family were illustrated using heatmaps (Figures 1A–M), while the dendrograms in each map represent hierarchical clustering of samples (OSCC tumor, normal) and genes (MMPs) based on their expression levels. Figure 1N displays the heatmap of significantly differentially expressed MMPs across the study datasets. To visualize common and dataset-specific differentially expressed MMPs, we have constructed an UpSet intersection diagram (Figure 2). The analysis successfully pinpointed six consistently upregulated MMPs in OSCC, specifically MMP-1, MMP-10, MMP-3, MMP-12, MMP-13, and MMP-9. The plot also demonstrates that, among all MMP family genes, only MMP-1 was upregulated in all the 13 datasets, followed by MMP-3 and MMP-10, which were overexpressed in 12 datasets. Other MMP family members exhibited high expression with partial overlap across the datasets, including MMP-12 in 11, MMP-13 in 8, and MMP-9 in 7 datasets. The observed intersection highlights consistent patterns of MMP dysregulation across OSCC datasets, justifying the prioritization of these core genes for further downstream analyses. Thus, these six highly upregulated MMPs may be utilized as potential diagnostic biomarkers for OSCC.

**FIGURE 1:**
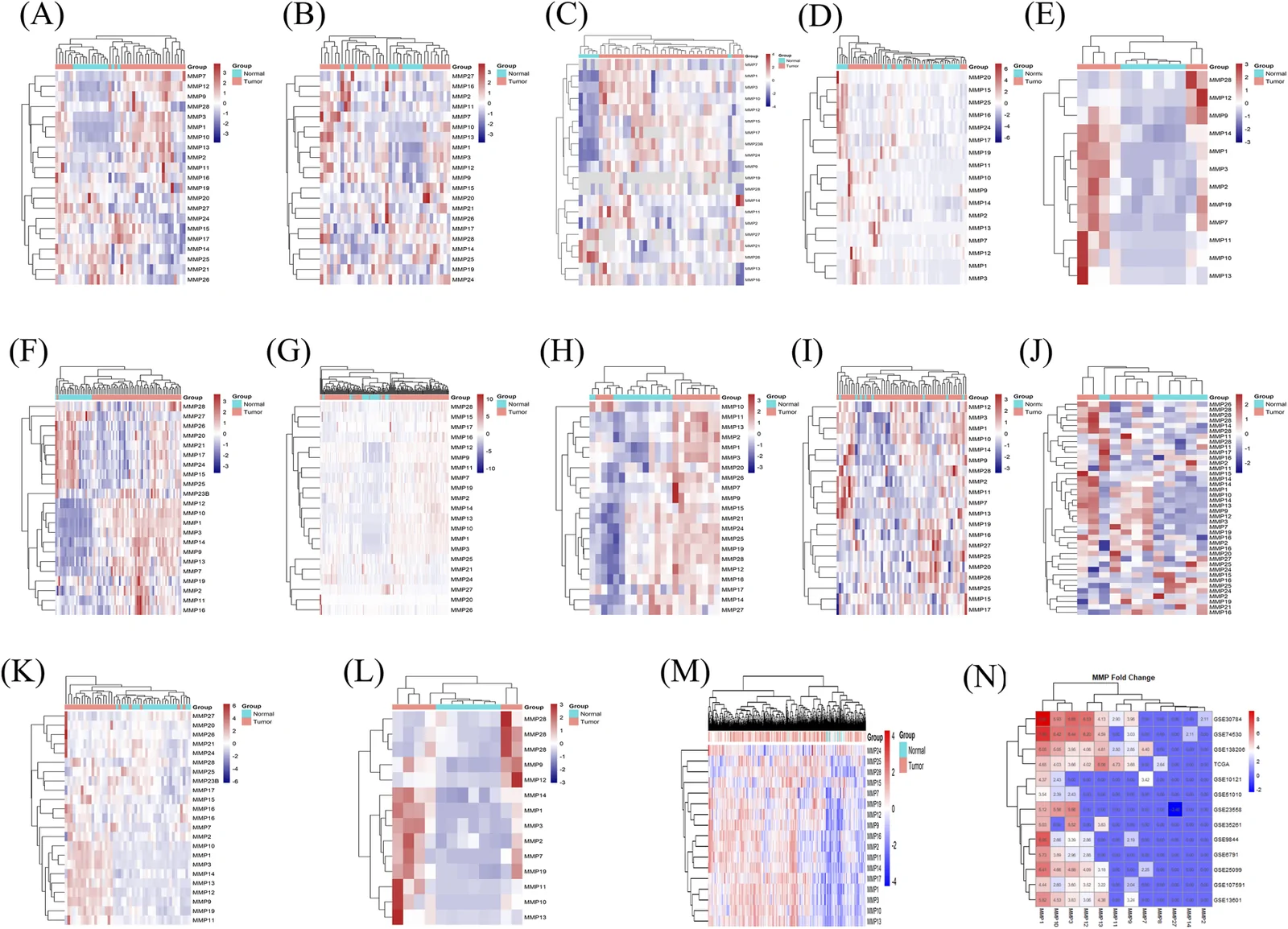
Heat map representation of differentially expressed MMP family genes between tumor and normal tissues in OSCC across the 13 datasets: **(A)** GSE6791, **(B)** GSE9844, **(C)** GSE10121, **(D)** GSE13601, **(E)** GSE23558, **(F)** GSE25099, **(G)** GSE30784, **(H)** GSE35261, **(I)** GSE51010, **(J)** GSE74530, **(K)** GSE107591, **(L)** GSE138206, **(M)** TCGA database, and **(N)** heatmap of significantly differentially expressed MMPs across the datasets. All the study datasets showed a FC of >2. The colour scale ranging from blue to red represents low to high levels of MMPs respectively in both tumor and normal samples.

**FIGURE 2:**
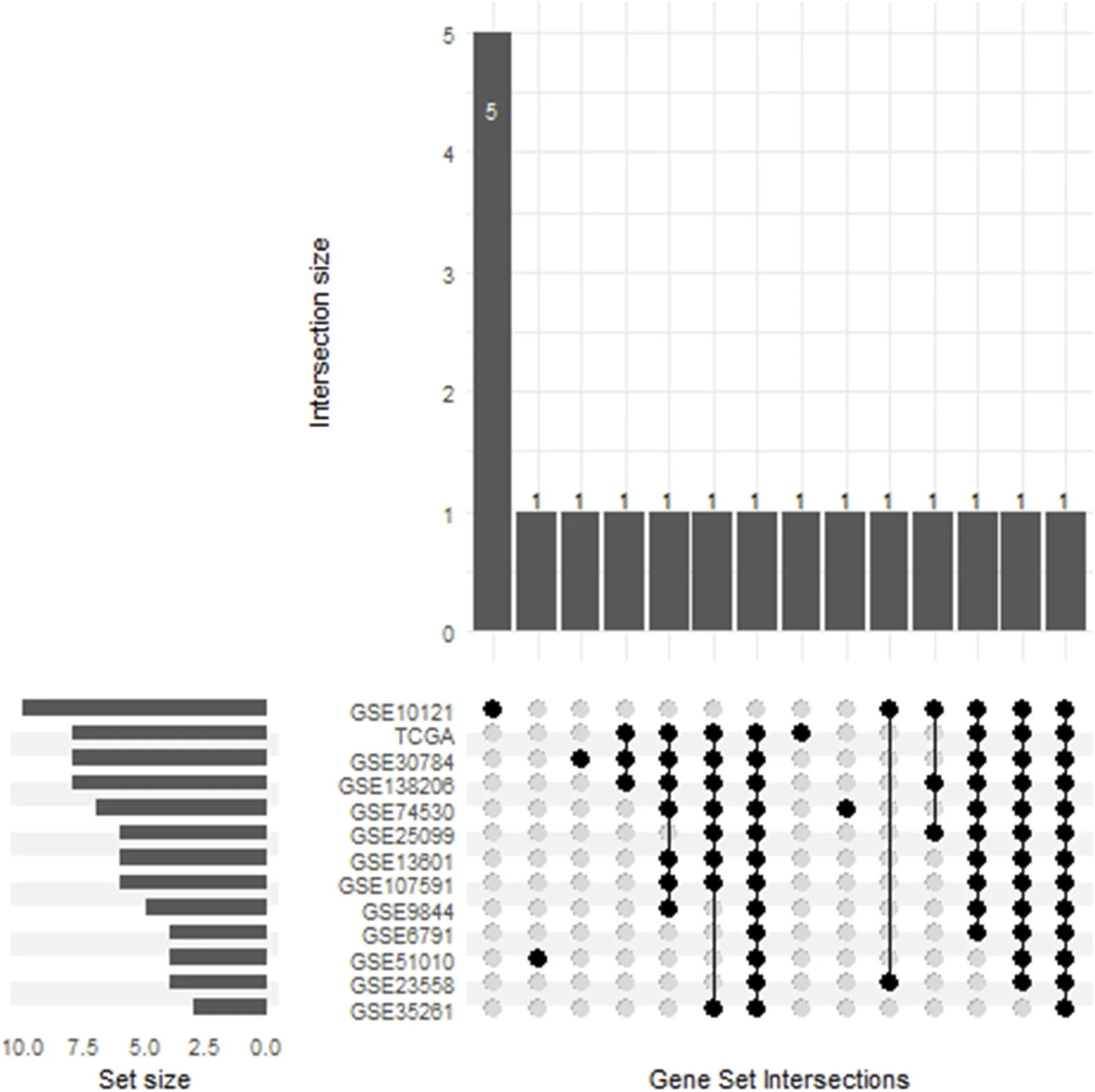
Overlapping MMPs across (12 GEO + TCGA) datasets were evaluated using an UpSet plot.

### 3.2 Biological pathway profiling in OSCC through GSEA

To assess the functional role of MMPs in OSCC tissues, GSEA was carried out according to the risk scores computed using the MMP-based signature model. GSEA results indicated that these genes strongly influence metabolic reprogramming in hallmark (h) and KEGG (c2) gene sets. Gene signatures were considered as strongly enriched based on a nominal *p*-value <0.05, False discovery rate (FDR) *q*-value <0.05, and NES values, representing enrichment strength and direction. As shown in Figure 3A, enrichment analysis using hallmark gene sets identified oxidative phosphorylation (NES = 2.7160, *p* = 1.00 × 10^−10^, FDR *q* = 4.17 × 10^−10^), fatty acid metabolism (NES = 2.0731, *p* = 7.58 × 10^−8^, FDR *q* = 2.23 × 10^−7^), adipogenesis (NES = 1.73, *p* = 2.47 × 10^−5^, FDR *q* = 5.89 × 10^−5^), and bile acid metabolism (NES = 1.71, *p* = 6.63 × 10^−4^, FDR *q* = 1.38 × 10^−3^) as top four highly active pathways. In KEGG gene sets, oxidative phosphorylation (hsa00190; NES = 2.66, *p* = 1.00 × 10^−10^, FDR *q* = 1.83 × 10^−9^), ribosomal pathway (hsa03010; NES = 2.64, *p* = 1.00 × 10^−10^, FDR *q* = 1.83 × 10^−9^), carbon metabolism (hsa01200; NES = 2.52, *p* = 1.00 × 10^−10^, FDR *q* = 1.83 × 10^−9^) and glyoxylate metabolism-related pathways (hsa00630; NES = 2.48, *p* = 2.61 × 10^−7^, FDR *q* = 3.13 × 10^−6^) were significantly upregulated (Figure 3B). Oncogenic signature analysis (Figure 3C) also demonstrated the upregulation of E2F3-transcripional pathways (E2F3_UP.V1_UP; NES = 1.55, *p* = 9.94 × 10^−4^, FDR *q* = 3.35 × 10^−3^), Serine/Threonine Kinase 33 (STK33)-associated signature pathways: STK33_SKM_DN (NES = 1.39, *p* = 3.85 × 10^−3^, FDR *q* = 3.95 × 10^−3^), STK33_NOMO_DN (NES = 1.39, *p* = 5.44 × 10^−3^, FDR *q* = 1.23 × 10^−2^), and NRF2-oxidative stress response pathways (NFE2L2. V2; NES = 1.33, *p* = 5.48 × 10^−3^, FDR *q* = 1.23 × 10^−2^), alongside dysregulated MMPs in OSCC tumor tissues.

**FIGURE 3:**
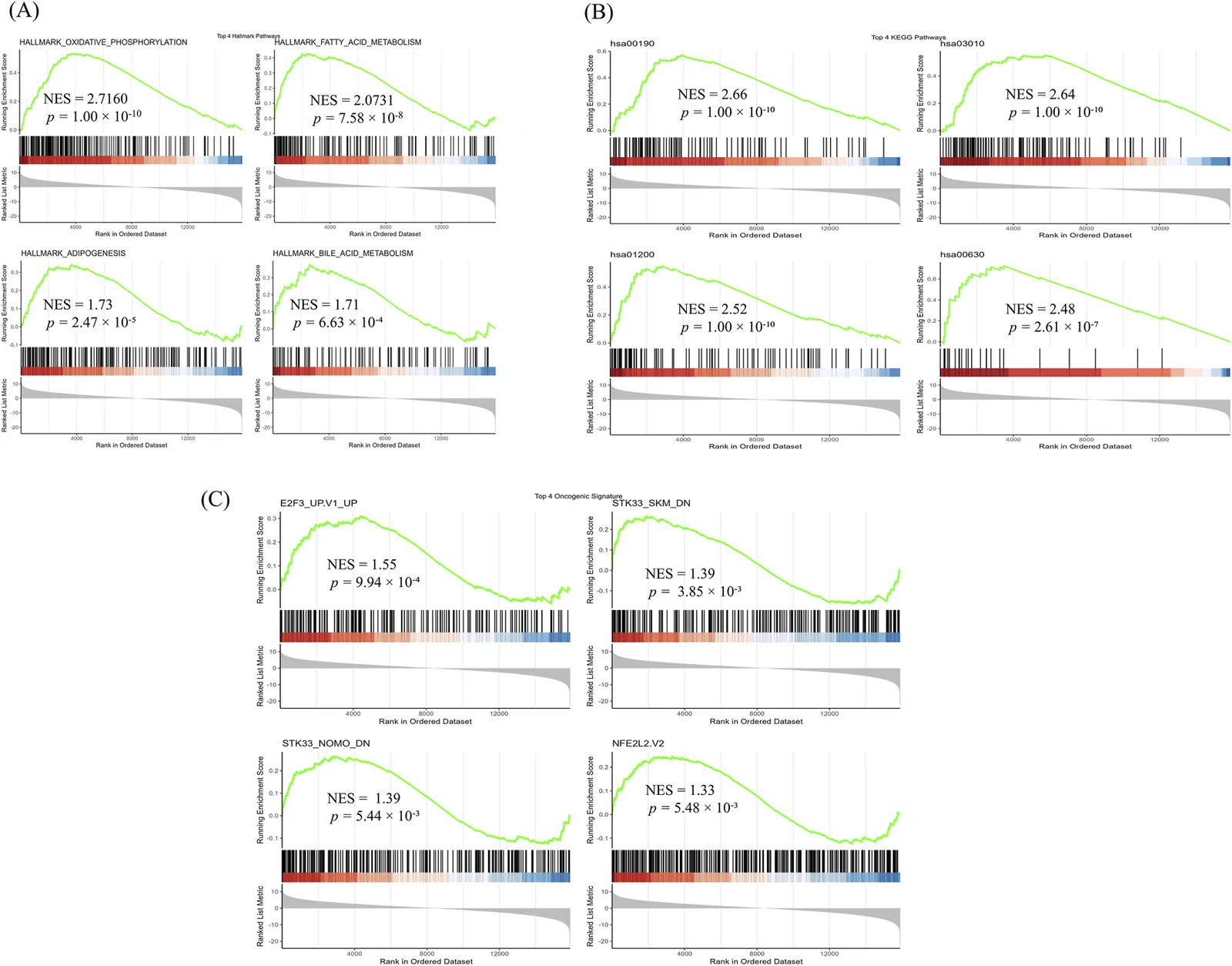
Enriched pathways based on **(A)** Hallmark gene sets, **(B)** KEGG gene sets, and **(C)** Oncogenic signature gene sets. Individual gene set names and KEGG pathway identifiers are shown in the respective panels.

### 3.3 Assessment of clinical data in OSCC patients using cox regression analysis

Clinical data of patients with OSCC from the TCGA database were utilized for Cox regression analysis as it provides most comprehensive clinicopathological parameters and largest OSCC tumor sample size (n = 258) among all the screened datasets. Univariate analysis revealed that tumor location (*p* = 0.0018), tobacco use (*p* = 0.0237), TNM stage III and IV (*p* = 0.0127), T stage (*p* = 0.0221), and N stage (*p* = 0.0127) were considerably related to patient’s survival rate (Figure 4A). Clinical parameters that exhibited significant association (*p* < 0.3) during the initial screening were included into a subsequent multivariate Cox regression analysis. After adjusting the effects of potential confounders, in final multivariate model, only tumor location (HR = 1.787, 95% confidence interval (CI) = 1.097–2.912, *p* = 0.0198) remained as an independent prediction factor in OSCC. However, tobacco use revealed a borderline significant relevance (HR = 1.525, 95% CI = 0.932–2.498, *p* = 0.0932), whereas TNM stage, T and N stages were not determined to be significant predictors of prognosis in the multivariate model (Figure 4B).

**FIGURE 4:**
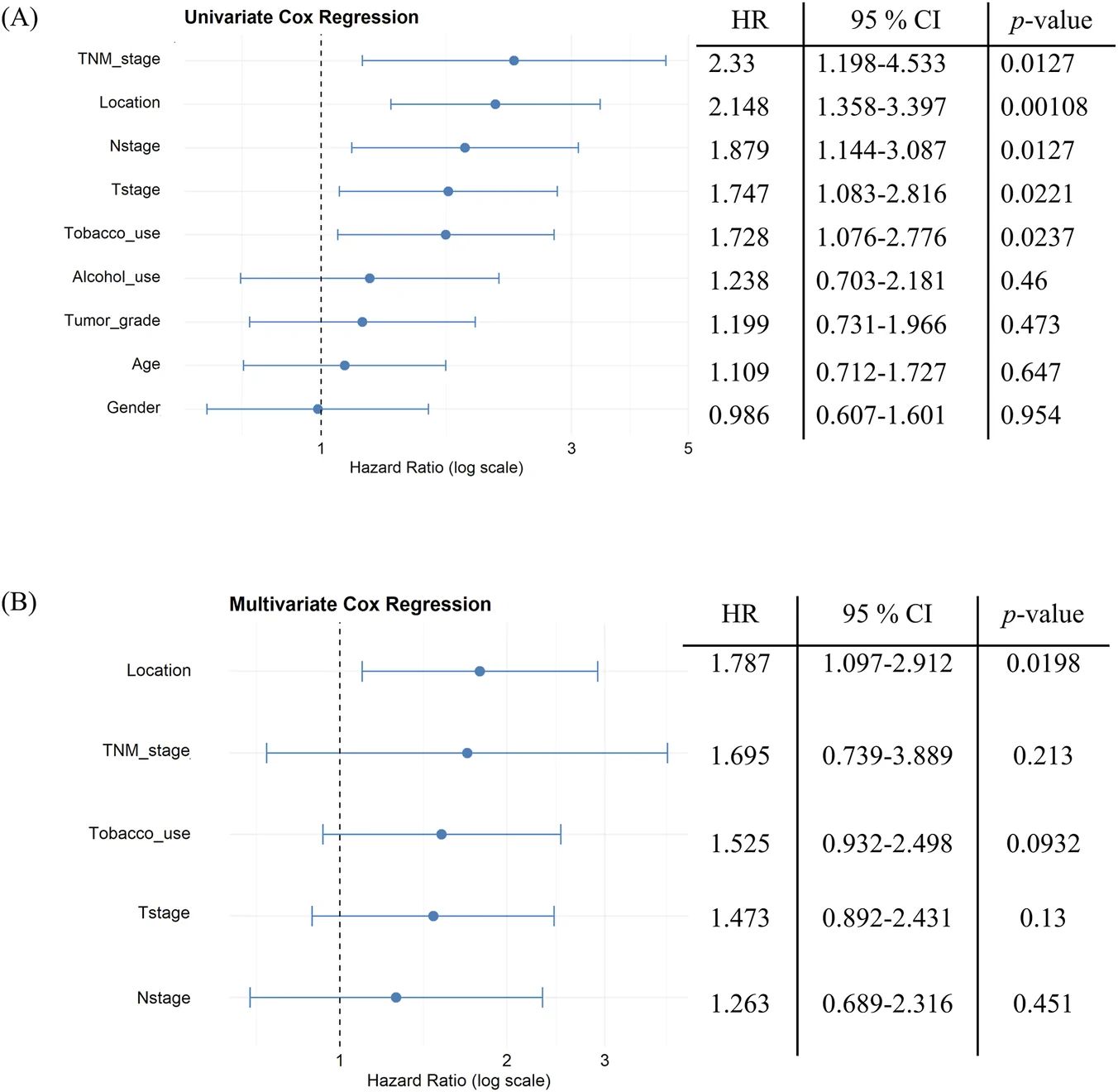
**(A)** Univariate forest plot and **(B)** Multivariate forest plot.

### 3.4 Survival analysis using MMP signature and TNM staging models for OSCC

To examine the prognostic significance of MMPs, DEGS were utilized to develop a prediction model using the clinical data from TCGA database. After Pearson’s correlation analysis, significantly expressed MMPs were integrated into the prognostic scoring model with corresponding hazard ratio (HR) and a 95% CI. The top six identified MMPs (MMP-1, MMP-10, MMP-3, MMP-12, MMP-13, and MMP-9) showed minimal clinical impact as independent prognostic factors (Figure 5A). None of the individual MMPs showed a statistically significant association with the OS outcome (HR = close to 1 and *p*-value >0.05) (Table 2). From the obtained MMPs forest plot, a nomogram was constructed using Cox proportional hazards to predict OS rates of OSCC patients (Figure 5B). To determine the predictive accuracy of MMP signature, ROC curve was generated. The C-index and Standard error (SE) were determined for standalone MMP signature model (C-index = 0.552, SE = 0.035), conventional TNM stage model (C-index = 0.585, SE = 0.031), and integrated combination model (C-index = 0.609, SE = 0.033) (Figure 6). AUC value of combined predictive model achieved 0.63, denoting a modest improvement in performance than that of individual MMP signature (AUC = 0.58) and TNM stage (AUC = 0.57) models. Subsequently, K-M survival analyses were implemented to determine the prognostic potential of both clinical TNM staging and MMP-based risk model. For TNM staging, the results revealed a non-significant association (*p* = 0.088) with OS probability (Figure 7A). Furthermore, OSCC patients were stratified into low- and high-risk cohorts using the median risk score as cut-off value to construct K-M survival curves based on the MMP signature (*p* = 0.045) (Figure 7B). Patients in high-risk groups demonstrated a considerably reduced survival probability, whereas stage II patients maintained a favourable, long-term survival probability. These findings suggest that risk distribution model of MMP signature provided effective prognostic-predictive factors for OSCC.

**FIGURE 5:**
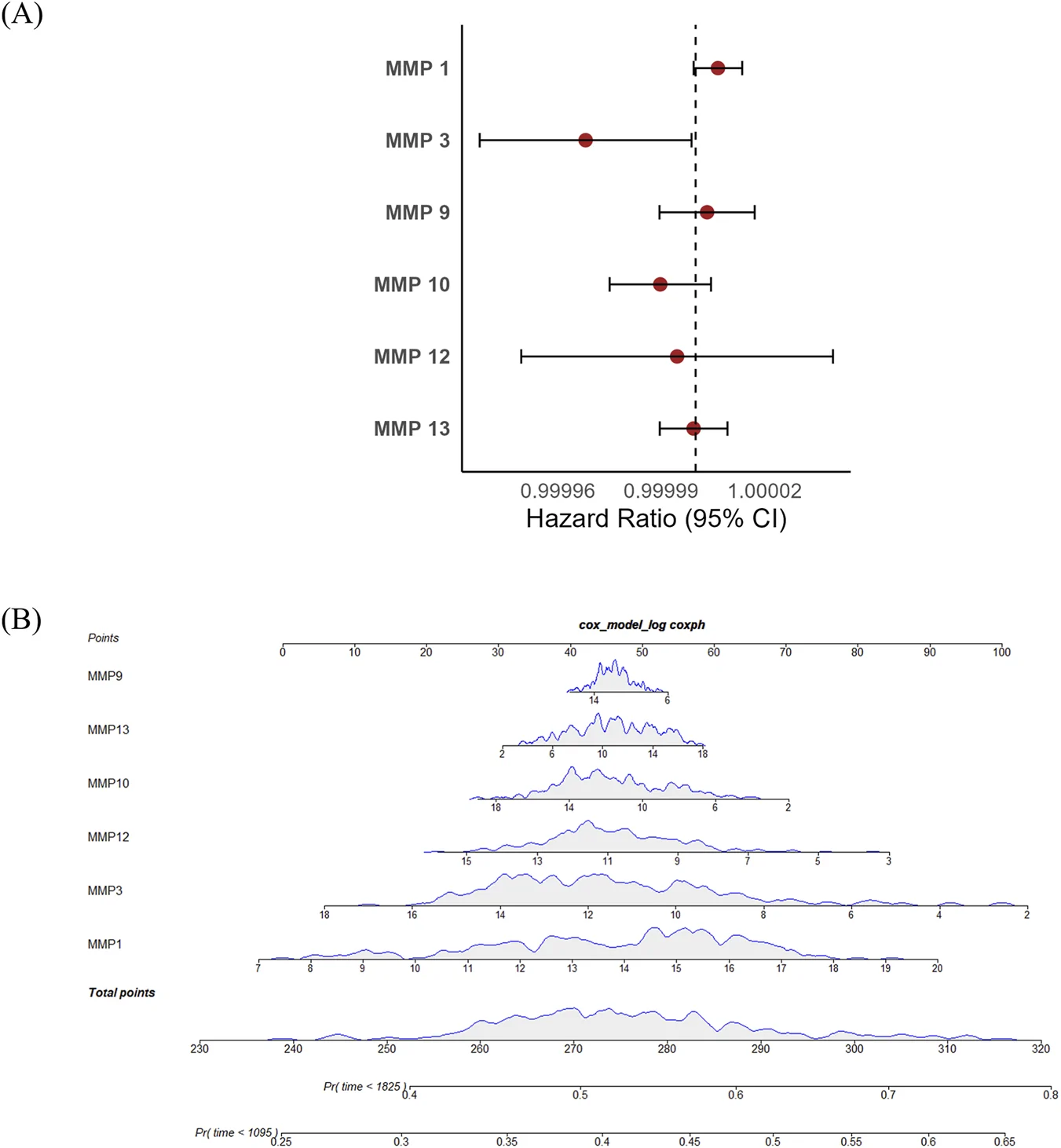
**(A)** Forest plot of MMPs included in the prediction model and **(B)** Nomogram of the MMP signature-based prediction model.

**TABLE 2:** Prognostic MMP signature model.

Gene	HR	Ci lower	Ci upper	*p*-value
MMP-1	1.000003	0.999998	1.000009	0.225149
MMP-3	0.999985	0.999962	1.000007	0.182221
MMP-9	1.000003	0.999992	1.000015	0.562269
MMP-10	0.999996	0.999988	1.000005	0.394388
MMP-12	0.999997	0.999968	1.000025	0.817345
MMP-13	1.000001	0.999993	1.000008	0.884995

**FIGURE 6:**
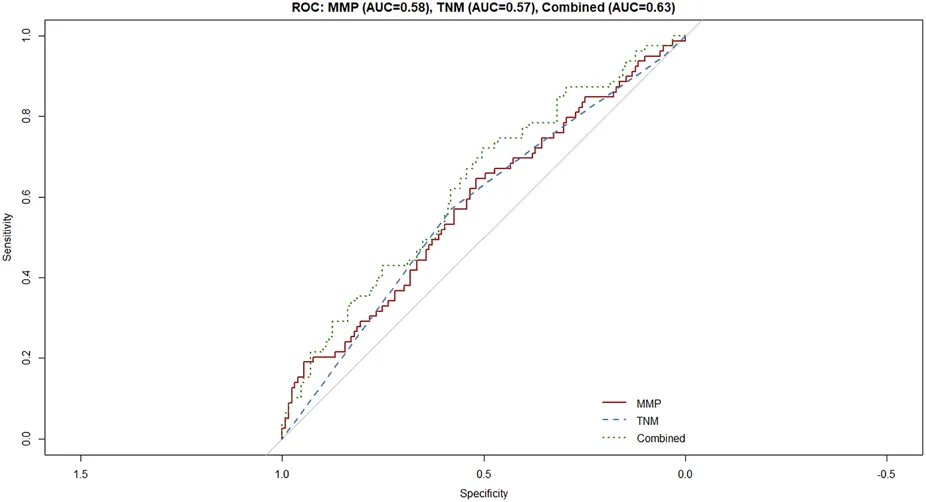
ROC curves of MMP signature, TNM staging models, and Combined models. C-index value for MMP-signature model is 0.552 (SE = 0.035), TNM stage is 0.585 (SE = 0.031), and combined model is 0.609 (SE = 0.033).

**FIGURE 7:**
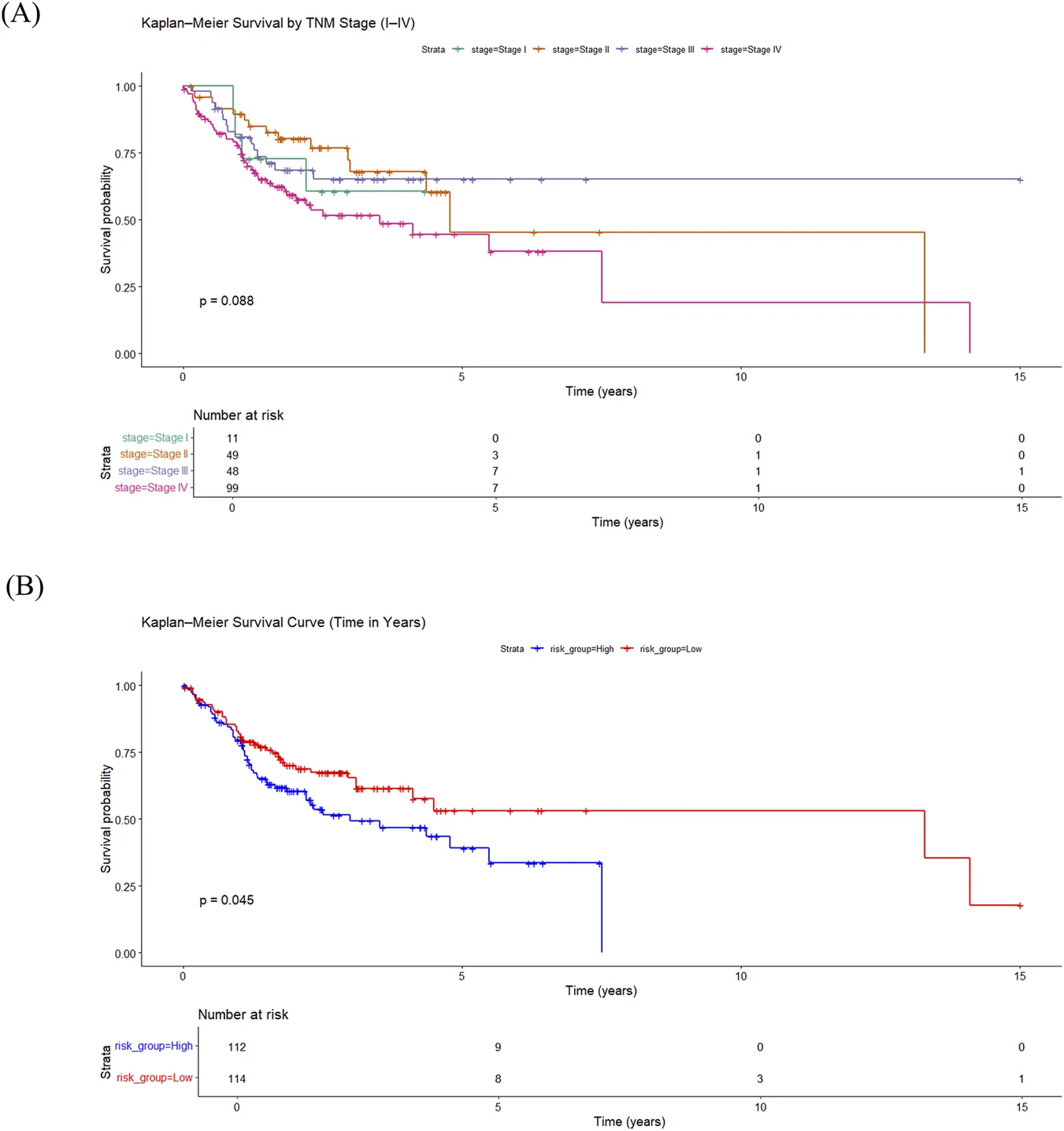
Kaplan-Meier survival analysis using **(A)** TNM Staging and **(B)** MMP signature.

### 3.5 Prognostic significance of integrated MMP signature and the TNM staging models

To achieve higher predictive accuracy, a prognostic nomogram was plotted by integrating the MMP signature with clinical TNM staging to predict survival outcomes (3-year and 5-year) (Figure 8A). ROC graphs were plotted to assess the nomogram’s predictive efficacy, yielding AUC values of 0.655 for 3-year survival and 0.612 for 5-year survival in OSCC patients (Figure 8B). The calibration graphs exhibited an excellent consistency between the predicted and observed clinical outcomes, confirming the strong predictive accuracy of the combined model (expression levels of six selected MMPs). Although the individual MMP genes were not found to be the independent prognostic factors in the Cox regression model, their combined expression profile contributed to patient risk stratification. Accordingly, the MMP-based risk score was calculated using the expression values of the selected MMP genes weighted by their Cox regression coefficients. In accordance with the obtained median risk score, OSCC cohort were categorized into two distinct groups (low- and high-risk) (Figure 8C). Evaluation of risk score patterns and patient’s survival status plots confirmed that the low-risk group patients exhibited a higher proportion of surviving patients and a lower mortality rate compared to the high-risk cohort. Consequently, 120 patients were reported to be alive and 105 patients approximately deceased (Figure 8D). Thus, the distribution map of survival outcomes showed that the high-risk group patients are associated with increased mortality rates. Therefore, the combined prediction model may offer clinicians a reliable approach to predict the prognosis rate of patients with OSCC.

**FIGURE 8:**
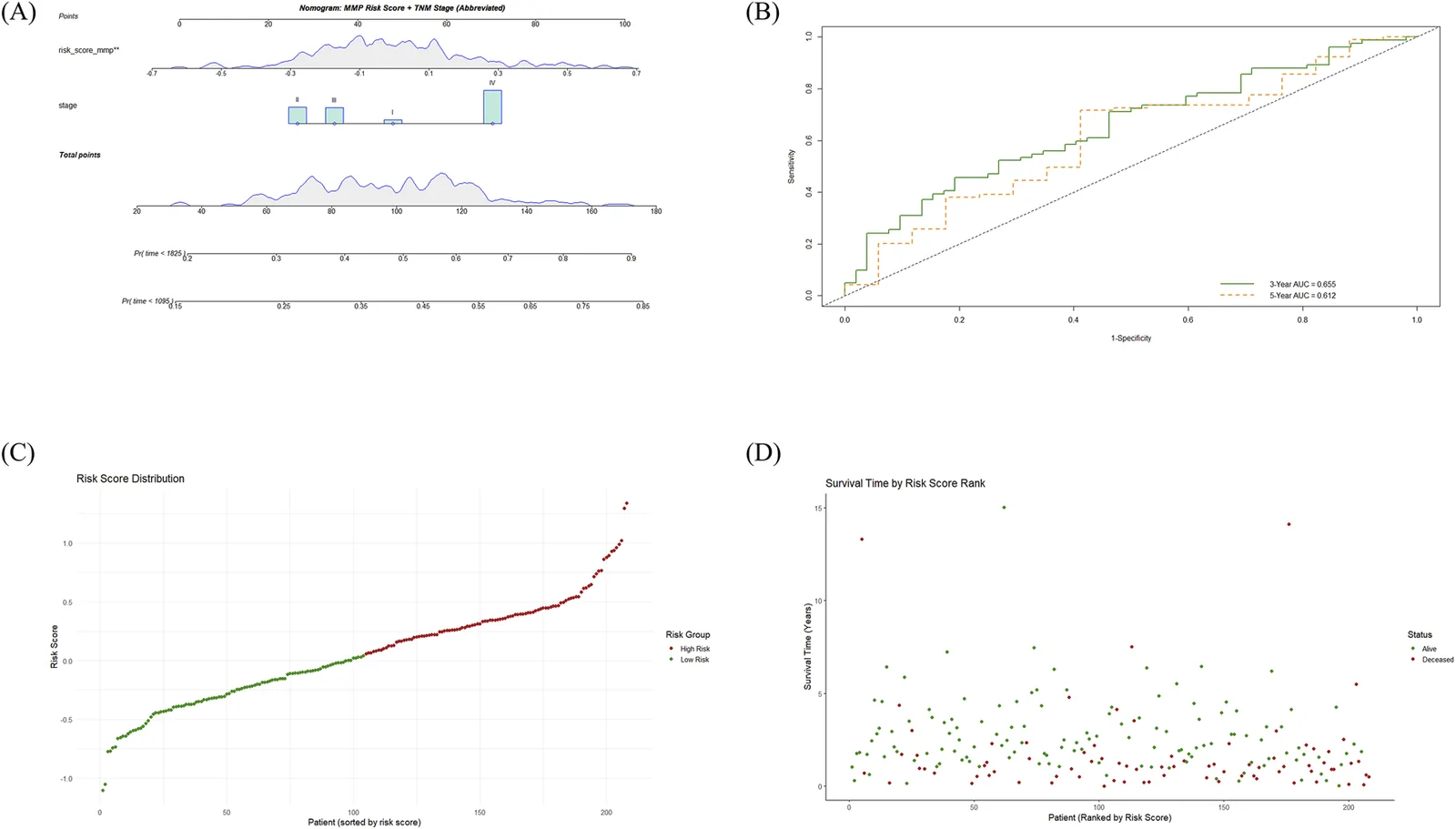
**(A)** MMP signature and TNM staging integrated prognostic nomogram to predict survival rate (3-year and 5-year), **(B)** ROC analysis of nomogram-based survival prediction (3-year and 5-year), **(C)** Risk-score distribution of OSCC patients classified by the MMP model. Green and red dots represent the low- and high-risk groups, **(D)** Survival outcomes of patients ranked by risk scores.

### 3.6 Evaluation of MMPs concentration in OSCC patients

To evaluate the serum concentration of MMPs, 79 OSCC patients (male - 59, female - 20) with confirmed diagnosis and no prior treatment were recruited. The control group included 91 subjects, consisting of 73 males and 18 females. Table 3 presents the clinicopathological characteristics of the patients evaluated for serum MMP-1, MMP-9, and MMP-10 levels. Among all OSCC cases, majority of the patients are exposed to oral habits such as betel quid chewing, smoking (87.43%), rest minor number of patients consume alcohol (12.65%). Serum concentrations of the three target MMPs (MMP-1, MMP-10, and MMP-9) showed a significant elevation in OSCC cases compared with control groups (*p* < 0.0001, *p* < 0.0001 and *p* = 0.0002) respectively (Figures 9A–C). The corresponding median values and results of two-tailed Mann-Whitney U test are summarized in Table 4. To evaluate the diagnostic capability of these three candidate MMPs for OSCC, ROC curve analysis was implemented. ROC analysis of serum MMP-1, MMP-10, and MMP-9 yielded AUC values of 0.8614, 0.8843, and 0.6653, respectively, which represents their promising diagnostic potential for OSCC screening (Figures 9D–F).

**TABLE 3:** Clinicopathological parameters of the OSCC patients.

Age	35–54	55–74
	55 (69.6%)	24 (30.4%)
Gender	Male	Female
	59 (74.68%)	20 (25.32%)
Body mass index	18.5–24.9 kg/m^2^	25–29.9 kg/m^2^
	74 (93.7%)	5 (6.32%)
TNM stages	Stages I – II	Stages III – IV
	49 (62.02%)	30 (37.97%)
Tumor site	Buccal mucosa	Tongue
	64 (81.01%)	15 (18.98%)
Symptoms	Ulcer/Swelling	Neck mass
	76 (96.2%)	2 (2.53%)
History of oral habits	Tobacco usage (Cigarette, betel nut chewing)	Alcohol intake
	69 (87.34%)	10 (12.65%)
Type of tumor growth	Ulcerative	Proliferative, infiltrative
	72 (91.13%)	7 (8.86%)

**FIGURE 9:**
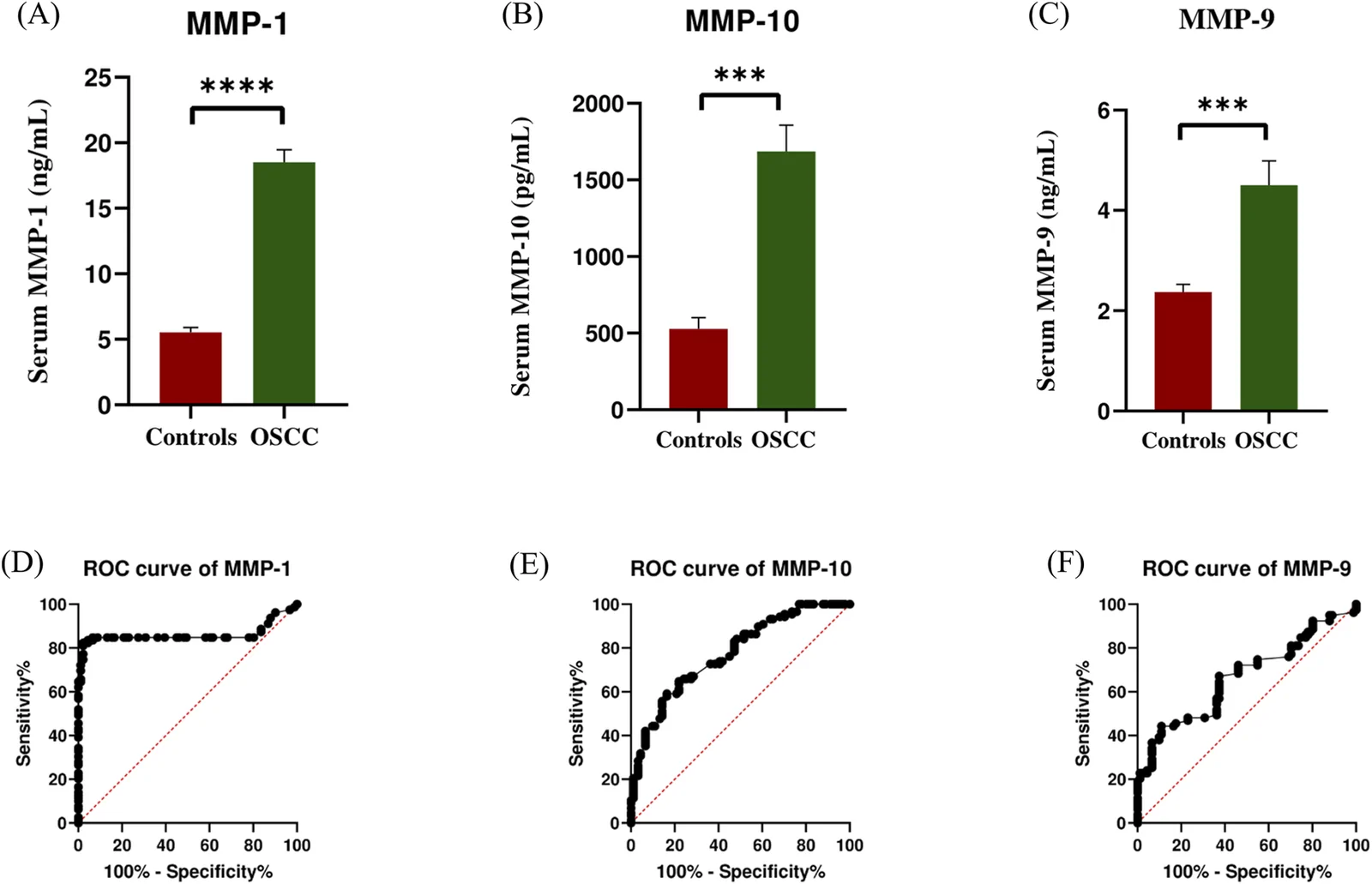
Serum MMP concentrations in control and OSCC cases. **(A)** MMP-1 levels, **(B)** MMP-10 levels, and **(C)** MMP-9 levels. The significant variations were assessed using non-parametric Mann-Whitney U test. The obtained p-value was indicated by ‘*’ where statistical significance, ‘****’ p < 0.0001 and ‘***’ p = 0.0002. ROC curve analysis were performed to evaluate diagnostic accuracy of serum **(D)** MMP-1, **(E)** MMP-10, and **(F)** MMP-9 levels between OSCC cases and control.

**TABLE 4:** Statistical comparison of serum MMP-1, MMP-10, and MMP-9 concentrations between OSCC patients and healthy controls.

Serum proteins	Controls (n = 91) Median concentrations (Mc)	OSCC (n = 79) Median concentrations (Mc)	Mann Whitney U	*p-*value
MMP-1	4.290 ng/mL	19.70 ng/mL	996.5	< 0.0001
MMP-10	259.5 pg/mL	1141 pg/mL	1799	< 0.0001
MMP-9	2.379 ng/mL	3.311 ng/mL	2406	0.0002

## 4 Discussion

Cancer is a genetic disease driven by DNA mutations that cause cells to divide uncontrollably. Because these genetic changes accumulate over many decades, cancer has emerged as the single largest obstacle to extending human life. Among all the distinct cancer types, OSCC remains as a deadly genetic disease, characterized by extensive intra- and inter-tumoral heterogeneity. This substantial cellular diversity leads to poor prognosis and therapeutic resistance against frontline chemotherapy treatments. The delayed diagnosis significantly reduces the survival duration of OSCC patients (Sahu et al., 2025), underscoring the pivotal role of biomarkers in early detection. The breakdown of ECM is a vital molecular step that enables cancer cells to spread to distant organs. In particular, members of the MMP family act as key proteolytic enzymes that degrade ECM components, strongly supporting their involvement in the onset and progression of multiple cancer types (Farina and Mackay, 2014; Zhang et al., 2014). Despite the roles of individual MMPs in OSCC progression are well-documented, it remains unclear how the combined expression profiles of the entire human MMP family impact overall patient survival. Much of the existing literature on MMP expression in OSCC focuses on an isolated subset of MMP genes via immunohistochemistry or salivary ELISA, yielding only a partial understanding of their role in oral tumor biology (Lawal et al., 2015; Shaukat et al., 2026). To address this gap, we systematically evaluated the transcriptomic patterns of the entire set of 23 identified human MMPs, using integrated bioinformatic approach, emphasizing the clinical relevance of their collective gene expression profiles. Subsequently, the obtained results were validated in OSCC patient’s serum samples of the South Indian population.

Numerous clinical and experimental studies have revealed an elevated MMP levels in OSCC tissues compared with normal tissues. For instance, Jordan et al. (2004) showed that the upregulated MMP-1 and MMP-9 expression in OSCC tissues were highly associated with OSCC progression (Jordan et al., 2004). Similarly, a study carried out in Nigeria demonstrated that the elevated immunohistochemical expression of MMP-2 directly correlates with a more aggressive OSCC phenotype (Lawal et al., 2015). In a recent research work, based on the immunohistochemical assays, the researchers observed the overexpression of MMP-1, MMP-3, MMP-9, and MMP-13 predominantly at advanced stages of OSCC (Saini et al., 2025). As documented in previous studies, our study also revealed the six top-most upregulated MMPs such as, MMP-1, MMP-10, MMP-3, MMP-12, MMP-13, and MMP-9 that are aberrantly expressed in 13 investigated datasets. Among the top six candidates, MMP-1 was upregulated in OSCC tumor tissues across all the evaluated 13 datasets. This was followed by MMP-10 and MMP-3 (each in 12 datasets), MMP-12 (11 datasets), MMP-13 (8 datasets), and MMP-9 (7 datasets), highlighting their collective potential as diagnostic biomarkers for OSCC. In line with previous studies, overexpression of MMPs are highly associated with metastatic spread and adverse prognosis of OSCC.

Metabolic reprogramming is the key emerging cancer hallmark that shapes TME to satisfy the high energy demands of tumor cells, thereby accelerating tumor cell proliferation and survival. In contrast to previous reports, focusing primarily on core signaling cascades, our finding indicates that upregulation of MMPs are also involved in metabolic reprogramming of oral tumor cells, suggesting enhanced energy production alongside biosynthetic support for ECM remodeling and invasion (Tan et al., 2020). The pathway enrichment analysis revealed three interconnected functional pathways which were found to be highly associated with top six dysregulated MMPs. First, the metabolic reprogramming was evident through activation of metabolism-related pathways and their subsequent influence on the expression of MMP family members (Waversen van et al., 2006). Second, the lipid-associated pathways including adipogenesis, fatty acid and bile acid metabolisms were highly active, indicating co-ordinated shift in tumor metabolic pathways. Although direct evidence linking these pathways with MMP activity in OSCC is limited, the identified metabolic and proliferation related pathways may be biologically interconnected with MMP-associated OSCC progression. Consistent with our computational findings, a previous study demonstrated that pyruvate kinase M2 enzyme driven glycolysis induces MMP expression, suggesting a direct mechanistic link between metabolic reprogramming and MMP-mediated cellular invasion in OSCC (Park et al., 2019). In addition to intrinsic factors, TME conditions actively drive metabolic reprogramming in OSCC. In line with this, Nakashima et al. (2020) revealed that hypoxia-induced cytosolic malic enzyme 1 transitions cellular metabolism from oxidative phosphorylation toward glycolysis, thereby elevating MMP levels and triggering oral tumor invasion. Beyond OSCC, studies across diverse malignancies confirm a clear link between these enriched pathways and MMP-mediated invasion as a conserved cancer hallmark. A supporting study by Li et al. (2020) reported that bile acids, such as cholic acid, stimulate MMP-9 expression in colon cancer. This occurs via the activation of mitogen-activated protein kinase, Activator protein 1 and Nuclear factor kappa-light-chain-enhancer of activated B cells signaling cascades, thereby inducing ECM degradation to drive invasion and metastatic dissemination. For example, MMP-9 upregulation is mechanically linked to the stimulation of E2F transcription factors, which further regulate cell cycle progression to promote cellular invasion and dissemination in non-small cell lung cancer (Johnson et al., 2012). Among the E2F family members, E2F3 plays a prominent role; its knockdown in brain tumor increased the rate of apoptosis and inhibited proliferation, invasive ability, and migration by affecting the expression of MMPs (MMP-2, MMP-3, MMP-9, and MMP-13). Mechanistically, E2F3 serves as a positive regulator of the MMP family. Upon E2F3 knockdown, the expression of MMP-2, MMP-3, MMP-9, and MMP-13 decreases, leading to diminished ECM degradation and the subsequent suppression of metastasis (Shen et al., 2017). In parallel, many studies emphasize that the elevated MMP gene expression promotes metastasis and modulates the oncogenic signaling pathways in various malignancies (Gobin et al., 2019; Chen et al., 2021; Luan et al., 2023; Shao et al., 2024).

To translate these findings into clinically useful predictions, prognostic modeling approaches, such as Cox proportional hazards model was employed to examine the survival outcomes in OSCC patients. Consequently, a robust survival prediction model was constructed utilizing all six identified dysregulated MMP genes. Utilizing TCGA database as a validation dataset, the univariate Cox regression analysis suggested that the TNM staging, tumor location, and tobacco usage were significantly associated with poor survival, highlighting their potential roles as independent prognostic factors in OSCC. However, to construct accurate prognostic model, we carried out the multivariate Cox regression analysis wherein majority of clinical variables lost their statistical significance. As exception, only the location of tumor showed stable association with the survival rate, indicating its ability as an independent prognostic factor. These findings suggest that the anatomical tumor location may influence OSCC survival outcomes independently of the conventional clinicopathological parameters. However, because the other clinical variables lost significance in the multivariate model, the prognostic role of tumor location should be interpreted cautiously and validated in larger independent cohorts.

Furthermore, Cox regression analysis was performed on the six top-most differentially expressed MMPs to assess their independent prognostic value. Based on the resulting MMP signature model, none of these MMP family genes emerged as independent and statistically significant prognostic factors. The comparative C-index analysis reinforces the prognostic benefit obtained by combining molecular and clinicopathological parameters. The individual MMP signature model demonstrated a relatively low concordance value, suggesting that although dysregulated MMP expression is associated with tumor-associated biological processes, the MMP gene signature alone has limited ability to predict OS of the OSCC patients. The standard TNM stage model achieved a slightly higher predictive accuracy, highlighting the well-established clinical relevance of anatomical tumor extent in prognostic assessment of OSCC. Notably, the integrated MMP-TNM stage model exhibited the moderate prognostic accuracy, indicating the improved risk stratification than either model alone. Despite the combined model revealed a modest improvement in concordance, this enhancement indicates the integrative role of molecular biomarkers with standard clinicopathological staging. These findings suggest that the MMP-related transcriptional changes may provide insights into tumor biology and metastatic potential beyond standard TNM staging. Therefore, the combined model offers a promising adjunctive approach for OSCC prognosis, particularly when integrated with existing staging approaches to enhance rather than replacing the current prognostic strategies. However, the moderate concordance values indicate the need for further validation across larger and diverse multicentre cohorts is required, prior to clinical implementation. Despite this, when integrated into a nomogram, these six MMP markers yield a cumulative risk score that effectively stratifies patients based on expected survival rates. Along with clinical prediction (nomogram), the molecular integration (ROC combined model = TNM stage + MMP markers) improved the predictive accuracy. Patients with advanced stages (III and IV) and elevated MMP scores increased the prediction probability of poor outcomes in survival rates. Collectively, these finding suggests that the OSCC prognosis relies on molecular-clinical integration rather than any single biomarker as an individual prognostic factor, thereby supporting the clinical utility of the developed nomogram and its underlying Cox model.

To validate the predicted MMPs as potential biomarkers, molecular-level detection was performed in the serum of patients with OSCC using ELISA. Our integrated computational analysis highlighted six candidate MMP biomarkers (MMP-1, MMP-10, MMP-3, MMP-12, MMP-13, and MMP-9) as significantly upregulated. From this group, we chose MMP-1, MMP-10, and MMP-9 for experimental validation. This selection was based on their robust consistency across multiple datasets and their well-established roles in extracellular matrix remodeling, basement membrane degradation, oral tumor invasion and progression (Stott-Miller et al., 2011; Caballero Escobar et al., 2016; Ghallab and Shaker, 2017; Monea and Pop, 2022; Shaukat et al., 2026). Based on this evidence the serum concentrations of MMP-1, MMP-10, and MMP-9 proteins were evaluated via ELISA in South Indian OSCC patients. The immunoassay results indicated a marked increase in the serum MMPs (MMP-1, MMP-10, and MMP-9) concentrations when comparing OSCC patients (n = 79) to healthy controls (n = 91). Previous studies have also confirmed the elevated expression levels of MMPs (MMP-1, MMP-2, MMP-3, MMP-9, and MMP-21) in OSCC patients across diverse global populations including Iranian, Taiwanese, Indian, Egyptian, and American cohort suggesting their potential role as oral tumor biomarkers (Stott-Miller et al., 2011; Tadbir et al., 2012; Chang et al., 2013; Ghallab and Shaker, 2017; Kherde and Gosavi, 2020; Baskaran et al., 2023; Benitha et al., 2023). Thus, our findings contribute to growing evidence, linking elevated MMP protein concentrations to oral tumor progression, particularly in treatment-naive cases. Consequently, all three validated MMPs (MMP-1, MMP-10, and MMP-9) can serve as minimally invasive prognostic biomarkers for OSCC in the South Indian cohort. To the best of our knowledge, we report the first study integrating computational prediction approaches with experimental validation of MMPs as potential biomarkers within South Indian OSCC patients. However, further research is crucial to fully elucidate the functional correlation among MMP family and their specific roles in oral tumor pathogenesis.

Nevertheless, the limitations of the present study should be acknowledged. The current bioinformatics approach was primarily based on retrospective gene expression data obtained from publicly-available repositories such as GEO and TCGA, which may contribute to heterogeneity across datasets and population-related bias. While the TCGA cohort provided clinicopathological data and survival information, it may not fully represent the South Indian OSCC cohort. Specifically, independent evaluation of gene expression profiles within the TCGA dataset indicated that no individual MMP family member possessed significant prognostic value. However, combining these top six upregulated MMPs yielded significant predictive efficacy than individual MMP which suggests greater clinical relevance. Furthermore, the modest predictive performance of the MMP-based model may serve as an adjunct to conventional clinicopathological parameters rather than a standalone prognostic tool. From the experimental perspective, the serum samples were used to explore the diagnostic potential of the identified candidate MMPs as minimally invasive biomarkers. However, the lack of matched tissue samples prevented tissue-level validation, representing a limitation of this study that demands further investigation. Also, the serum validation was performed only for three candidate MMPs (MMP-1, MMP-10, and MMP-9), while MMP-3, MMP-12, and MMP-13 were not experimentally validated. Furthermore, this study evaluated the diagnostic potential of candidate MMPs rather than their therapeutic efficacy. Despite their biological positioning as potential therapeutic candidates in OSCC, further functional and clinical investigations are required to confirm their therapeutic relevance. Thus, future studies involving experimental validation in large independent patient cohorts and multicentre patients are necessary to assess the role of MMPs as potential clinical biomarkers.

## 5 Conclusion

Despite tremendous progress in OSCC screening, the mortality rate remain high due to late diagnosis, often means the disease has already metastasized. The current study utilized an integrated approach to predict and validate the functional roles of MMP family genes in OSCC. An integrated transcriptomics approach reveals that a set of MMPs such as MMP-1, MMP-10, MMP-3, MMP-12, MMP-13, and MMP-9 are upregulated in OSCC tissue samples. Functional enrichment analysis revealed the key genes and pathways involved in OSCC pathogenesis. In conclusion, the experimental serum validation of this study reinforces the link between oral tumor progression with the elevated MMP protein levels (MMP-1, MMP-10, and MMP-9), particularly in treatment-naive OSCC patients. These findings suggest that MMP-1, MMP-10, and MMP-9 serve as promising candidate biomarkers for detection of OSCC, suggesting their potential utility as minimally invasive biomarkers in South Indian cohort. However, future research is necessary to investigate the mechanisms responsible for oral tumor invasion and its potential implications in disease diagnosis, prognosis and treatment of OSCC.

## Data Availability

The datasets presented in this study can be found in online repositories. The names of the repository/repositories and accession number(s) can be found in the article/supplementary material.

## References

[B1] Anwar S. Pratap P. D. (2023). The interplay of matrix metalloproteases in oral cancer and their associated clinical outcomes. Int. J. Curr. Res. Life Sci. 12, 3465–3470.

[B2] Barrett T. Suzek T. O. Troup D. B. Wilhite S. E. Ngau W. C. Ledoux P. (2005). NCBI GEO: mining millions of expression profiles - database and tools. Nucleic Acids Res. 33, 562–566. 10.1093/nar/gki022 15608262 PMC539976

[B3] Baskaran A. Santhadevy A. Vezhavendhan N. Sivaramakrishnan M. Santhanam V. L. Suganya R. (2023). Expression of matrix Metalloproteinase-21 in oral squamous cell carcinoma. Cureus 15, 1–5. 10.7759/cureus.34256 36855499 PMC9968393

[B4] Benitha G. Ramani P. Jayaraman S. R A. Ramalingam K. Krishnan M. (2023). Evaluation of serum levels of matrix MetalloProteinase-9 (MMP-9) in oral squamous cell carcinoma and its clinicopathological correlation. Cureus 9. 10.7759/cureus.34954 36938194 PMC10018444

[B5] Caballero Escobar C. Cabezas Mojon J. Serfatty Castro G. Loughney Gonzalez A. Fernandez Dominguez M. (2016). A review on salivary biomarkers for oral cancer detection. Oral Patol. Oral Y Cir. Bucal, S49–S50. 10.4317/medoral.17644119

[B6] Chang P. Y. Kuo Y. B. Wu T. L. Liao C. T. Sun Y. C. Yen T. C. (2013). Association and prognostic value of serum inflammation markers in patients with leukoplakia and oral cavity cancer. Clin. Chem. Lab. Med. 51, 1291–1300. 10.1515/cclm-2012-0504 23154424

[B7] Chen W. Huang S. Shi K. Yi L. Liu Y. Liu W. (2021). Prognostic role of matrix metalloproteinases in cervical cancer: a meta-analysis. Cancer control. 28, 1–13. 10.1177/10732748211033743 34482737 PMC8424604

[B8] Farina A. R. Mackay A. R. (2014). Gelatinase B/MMP-9 in tumour pathogenesis and progression. Cancers (Basel) 6, 240–296. 10.3390/cancers6010240 24473089 PMC3980597

[B9] Ferlay J. Rebecca M. E. Mph L. S. (2024). Global cancer statistics 2022: GLOBOCAN estimates of incidence and mortality worldwide for 36 cancers in 185 countries. A Cancer J. Clin. 74, 229–263. 10.3322/caac.21834 38572751

[B10] Gangane N. M. Ghongade P. V. Patil B. U. Atram M. (2024). Oral cavity cancer incidence and survival trends: a population-based study. J. Cancer Res. Ther. 20, 1446–1452. 10.4103/jcrt.jcrt_2720_22 38261454

[B11] Ghallab N. A. Shaker O. G. (2017). Serum and salivary levels of chemerin and MMP-9 in oral squamous cell carcinoma and oral premalignant lesions. Clin. Oral Investig. 21, 937–947. 10.1007/s00784-016-1846-8 27161218

[B12] Gobin E. Bagwell K. Wagner J. Mysona D. Sandirasegarane S. Smith N. (2019). A pan-cancer perspective of matrix metalloproteases (MMP) gene expression profile and their diagnostic/prognostic potential. BMC Cancer 19, 1–10. 10.1186/s12885-019-5768-0 31200666 PMC6567474

[B13] Herrero R. Castellsagu X. Pawlita M. Lissowska J. Kee F. Balaram P. (2003). Human papillomavirus and oral cancer: the international agency for research on cancer multicenter study. 95. 10.1093/jnci/djg107 14652239

[B14] Jena D. Padhi B. K. Zahiruddin Q. S. Ballal S. Kumar S. Bhat M. (2024). Estimation of Burden of Cancer Incidence and Mortality in India: Based on Global Burden of Disease Study 1990 – 2021. 10.1186/s12885-024-13035-6 PMC11476122 39407152

[B15] Johnson J. L. Pillai S. Pernazza D. Sebti S. M. Lawrence N. J. Chellappan S. P. (2012). Regulation of matrix metalloproteinase genes by E2F transcription factors: rb – raf-1 interaction as a novel target for metastatic disease. Ther. Targets, Chem. Biol. 72, 516–526. 10.1158/0008-5472.CAN-11-2647 22086850 PMC3261351

[B16] Jordan R. C. K. Macabeo-Ong M. Shiboski C. H. Dekker N. Ginzinger D. G. Wong D. T. W. (2004). Overexpression of matrix metalloproteinase-1 and -9 mRNA is associated with progression of oral dysplasia to cancer. Clin. Cancer Res. 10, 6460–6465. 10.1158/1078-0432.CCR-04-0656 15475433

[B17] Kessenbrock K. Piaks V. Werb Z. (2010). Matrix metalloproteinases: regulators of the tumor microenvironment. Cell 141, 1–7. 10.1016/j.cell.2010.03.015.Matrix PMC2862057 20371345

[B18] Kherde P. Gosavi S. (2020). Serum levels of matrix metalloproteinase-2 in patients with oral submucous fibrosis and oral squamous cell carcinoma. Int. J. Res. Med. Sci. 8, 636. 10.18203/2320-6012.ijrms20200248

[B19] Kolegova E. S. Korobeynikova A. A. Vorobev R. S. Fedorova I. K. Kulbakin D. E. Kononova G. A. (2025). Genetic alterations of oral cancer associated with progression in young adults. Russ. J. Genet. 61, 564–572. 10.1134/S1022795425700085

[B20] Lawal A. O. Adisa A. O. Kolude B. Adeyemi B. F. (2015). Immunohistochemical expression of MMP-2 and MMP-8 in oral squamous cell carcinoma. J. Clin. Exp. Dent. 7, e203–e207. 10.4317/jced.52047 26155333 PMC4483324

[B21] Leek J. T. Johnson W. E. Parker H. S. Jaffe A. E. Storey J. D. (2012). The SVA package for removing batch effects and other unwanted variation in high-throughput experiments. Bioinformatics 28, 882–883. 10.1093/bioinformatics/bts034 22257669 PMC3307112

[B54] Li S. Ung T. T. Nguyen T. T. Sah D. K. Park S. Y. (2020). Cholic acid stimulates MMP-9 in human colon cancer cells via activation of MAPK, AP-1, and NF-κB activity. Int. J. Mol. Sci. 21, 1–16. 32408577 10.3390/ijms21103420 PMC7279292

[B22] Li C. Dong X. Li B. (2024). Tumor microenvironment in oral squamous cell carcinoma. Front. Immunol. 15, 1–14. 10.3389/fimmu.2024.1485174 39744628 PMC11688467

[B23] Liberzon A. Birger C. Thorvaldsdóttir H. Ghandi M. Mesirov J. P. Tamayo P. (2015). The molecular signatures database hallmark gene set collection. Cell Syst. 1, 417–425. 10.1016/j.cels.2015.12.004 26771021 PMC4707969

[B24] Luan H. Jian L. Huang Y. Guo Y. Zhou L. (2023). Identification of novel therapeutic target and prognostic biomarker in matrix metalloproteinase gene family in pancreatic cancer. Sci. Rep. 13, 1–14. 10.1038/s41598-023-44506-8 37821678 PMC10567842

[B25] Lucas F. A. S. Fowler J. Chang K. Kopetz S. Vilar E. Scheet P. (2014). Cancer *in silico* drug discovery: a systems biology tool for identifying candidate drugs to target speci fi c molecular tumor subtypes. Model. Tech. 13, 3230–3240. 10.1158/1535-7163.MCT-14-0260 PMC4341901 25349306

[B26] Miranda-filho A. Bray F. (2020). Global patterns and trends in cancers of the lip, tongue and mouth. Oral Oncol. 102, 104551. 10.1016/j.oraloncology.2019.104551 31986342

[B27] Monea M. Pop A. M. (2022). The use of salivary levels of matrix metalloproteinases as an adjuvant method in the early diagnosis of oral squamous cell carcinoma: a narrative literature review. Curr. Issues Mol. Biol. 44, 6306–6322. 10.3390/cimb44120430 36547091 PMC9776994

[B28] Muthu K. Vaishnavi V. Sivadas G. (2018). Warning signs and symptoms of oral cancer and its differential. Diagnosis 10, 138–143. 10.5530/jyp.2018.10.32

[B53] Nakashima C. Kirita T. Yamamoto K. Mori S. Luo Y. Sasaki T. (2020). Malic enzyme 1 is associated with tumor budding in oral squamous cell carcinomas. Int. J. Mol. Sci. 21, 1–13. 10.3390/ijms21197149 32998265 PMC7582746

[B29] Niklander S. E. (2021). Inflammatory mediators in oral cancer: pathogenic mechanisms and diagnostic potential. Front. Oral Heal. 2, 642238. 10.3389/froh.2021.642238 35047997 PMC8757707

[B30] Park Y. J. Kim J. Y. Lee D. Y. Zhang X. Bazarsad S. Chung W. Y. (2019). PKM2 enhances cancer invasion via ETS-1dependent induction of matrix metalloproteinase in oral squamous cell carcinoma cells. PLoS One 14, 1–17. 10.1371/journal.pone.0216661 31071178 PMC6508653

[B31] Patel B. Shah S. Shukla S. Shah P. Patel P. S. (2007). Clinical significance of MMP-2 and MMP-9 in patients with oral cancer. J. Sci. Spec. Head Neck 29, 564–572. 10.1002/hed.20561 17252594

[B32] Rahadiani N. Habiburrahman M. Stephanie M. Handjari D. R. Krisnuhoni E. (2023). Estimated projection of oral squamous cell carcinoma annual incidence from twenty years registry data: a retrospective cross-sectional study in Indonesia. PeerJ 11, 1–31. 10.7717/peerj.15911 37663292 PMC10473041

[B33] Ray J. G. Ganguly M. Rao B. S. Mukherjee S. Mahato B. Chaudhuri K. (2013). Clinico - epidemiological profile of oral potentially malignant and malignant conditions among areca nut, tobacco and alcohol users in eastern India: a hospital based study. J. Oral Maxillofac. Pathol. 17, 1–6. 10.4103/0973-029x.110720 23798829 PMC3687188

[B34] Ritchie M. E. Phipson B. Wu D. Hu Y. Law C. W. Shi W. (2015). Limma powers differential expression analyses for RNA-Sequencing and microarray studies. Nucleic Acids Res. 43, e47. 10.1093/nar/gkv007 25605792 PMC4402510

[B35] Robinson M. D. McCarthy D. J. Smyth G. K. (2009). edgeR: a bioconductor package for differential expression analysis of digital gene expression data. Bioinformatics 26, 139–140. 10.1093/bioinformatics/btp616 19910308 PMC2796818

[B36] Rutkowska M. Hnitecka S. Nahajowski M. Dominiak M. Gerber H. (2020). Oral Cancer: The First Symptoms and Reasons for Delaying Correct Diagnosis and Appropriate Treatment. 10.17219/acem/116753 32598579

[B52] Sahu G. C. Shankar R. Singhavi H. R. Daga D. Rai D. K. Gupta M. (2025). Delay in the diagnosis of oral cancer in India, time to focus toward capacity building of physicians: A prospective observational study. Indian J. Cancer 62, 128–134. 10.4103/ijc.ijc_44_22 40377616

[B37] Saini J. Bakshi J. Panda N. K. Sharma M. Yadav A. K. Kamboj K. (2024). Serum concentration of MMP-9 as a predictive biomarker for the progression of oral cancer. J. Maxillofac. Oral Surg. 23, 1079–1088. 10.1007/s12663-023-01932-5 39376770 PMC11455997

[B38] Saini J. Bakshi J. Panda N. K. Sharma M. Yadav A. K. Narayansami S. (2025). Diagnostic and prognostic accuracy of MMPs and TIMPs in oral cancer patients on enzyme-linked immunosorbent assay (ELISA) as compared to immunohistochemistry (IHC). Indian J. Surg. Oncol. 16, 601–610. 10.1007/s13193-024-02113-7 40337044 PMC12052617

[B39] Shao L. Liu W. Zhang C. Ma W. Yu X. Han J. (2024). The role and function of secretory protein matrix Metalloproteinase-3 (MMP3) in cervical cancer. Iran. J. Public Health 53, 855–866. 10.18502/ijph.v53i4.15562 39444485 PMC11493579

[B40] Shaukat A. Nisar S. Asghar M. Kaleem M. Zafar M. S. (2026). MMP-9 as a diagnostic salivary biomarker for early detection of oral cancers: systematic review and meta-analysis. BMC Oral Health 26, 219. 10.1186/s12903-025-07513-x 41484977 PMC12866002

[B41] Shen Z. Liu X. Chen C. Lu J. (2017). Knockdown of E2F3 inhibits proliferation, migration, and invasion and increases apoptosis in glioma cells. Oncol. Res. 25, 1555–1566. 10.3727/096504017X14897158009178 28337965 PMC7841128

[B42] Stott-Miller M. Houck J. R. Lohavanichbutr P. Méndez E. Upton M. P. Futran N. D. (2011). Tumor and salivary matrix metalloproteinase levels are strong diagnostic markers of oral squamous cell carcinoma. Cancer Epidemiol. Biomarkers Prev. 20, 2628–2636. 10.1158/1055-9965.EPI-11-0503 21960692 PMC3237810

[B43] Tadbir A. A. Purshahidi S. Ebrahimi H. Khademi B. Malekzadeh M. Mardani M. (2012). Serum level of MMP-3 in patients with oral squamous cell carcinoma - lack of association with clinico-pathological features. Asian Pac. J. Cancer Prev. 13, 4545–4548. 10.7314/APJCP.2012.13.9.4545 23167377

[B44] Tan B. Jaulin A. Bund C. Hassiba O. Wendling C. Chenard M.-P. (2020). Matrix Metalloproteinase-11 promotes early mouse signaling pathway. Enhanc. ER Stress 1–20. 10.3390/cancers12092357 PMC7565046 32825455

[B45] Thavarool S. B. Muttath G. Nayanar S. Duraisamy K. Bhat P. Shringarpure K. (2019). Improved survival among oral cancer patients: findings from a retrospective study at a tertiary care cancer centre in rural Kerala, India. World J. Surg. Oncol. 17, 1–7. 10.1186/s12957-018-1550-z 30635062 PMC6330462

[B46] Visse R. Nagase H. (2003). Matrix metalloproteinases and tissue inhibitors of metalloproteinases: structure, function, and biochemistry. Circ. Res. 92, 827–839. 10.1161/01.RES.0000070112.80711.3D 12730128

[B47] Walsh L. A. Alvarez M. J. Sabio E. Y. Vahdat L. T. Califano A. Chan T. A. (2017). An Integrated Systems Biology Approach Identifies TRIM25 as a Key Determinant of Breast Cancer Article an Integrated Systems Biology Approach Identifies TRIM25 as a Key Determinant of Breast Cancer Metastasis, 1623–1640. 10.1016/j.celrep.2017.07.052 PMC5985663 28813674

[B48] Waversen van C. Sun Y. Cheung H. Moraes C. (2006). Oxidative phosphorylation dysfunction modulates expression of extracellular matrix—remodeling genes and invasion. Carcinogenesis 27, 409–418. 10.1093/carcin/bgi242 16221732 PMC2778475

[B49] Weinstein J. N. Collisson E. A. Mills G. B. Shaw K. R. M. Ozenberger B. A. Ellrott K. (2013). The cancer genome atlas pan-cancer analysis project. Nat. Genet. 45, 1113–1120. 10.1038/ng.2764 24071849 PMC3919969

[B50] Yang J. J. Yu D. Wen W. Shu X. Saito E. Rahman S. (2019). Tobacco smoking and mortality in Asia A pooled meta-analysis. JAMA Netw. Open 2, 1–14. 10.1001/jamanetworkopen.2019.1474 30924901 PMC6450311

[B51] Zhang G. Miyake M. Lawton A. Goodison S. Rosser C. J. (2014). Matrix metalloproteinase-10 promotes tumor progression through regulation of angiogenic and apoptotic pathways in cervical tumors. BMC Cancer 14, 1–14. 10.1186/1471-2407-14-310 24885595 PMC4022983

